# Tool Wear State Identification Based on SVM Optimized by the Improved Northern Goshawk Optimization

**DOI:** 10.3390/s23208591

**Published:** 2023-10-20

**Authors:** Jiaqi Wang, Zhong Xiang, Xiao Cheng, Ji Zhou, Wenqi Li

**Affiliations:** 1 School of Mechanical Engineering, Zhejiang Sci-Tech University, Hangzhou 310018, China; 202130605293@mails.zstu.edu.cn (J.W.); xz@zstu.edu.cn (Z.X.); 202120601079@mails.zstu.edu.cn (J.Z.); 202120601026@mails.zstu.edu.cn (W.L.); 2 Longgang Institute of Zhejiang Sci-Tech University, Wenzhou 325802, China

**Keywords:** tool wear state identification, recursive feature elimination, improved northern goshawk optimization, support vector machine

## Abstract

Tool wear condition significantly influences equipment downtime and machining precision, necessitating the exploration of a more accurate tool wear state identification technique. In this paper, the wavelet packet thresholding denoising method is used to process the acquired multi-source signals and extract several signal features. The set of features most relevant to the tool wear state is screened out by the support vector machine recursive feature elimination (SVM-RFE). Utilizing these selected features, we propose a tool wear state identification model, which utilizes an improved northern goshawk optimization (INGO) algorithm to optimize the support vector machine (SVM), hereby referred to as INGO-SVM. The simulation tests reveal that INGO demonstrates superior convergence efficacy and stability. Furthermore, a milling wear experiment confirms that this approach outperforms five other methods in terms of recognition accuracy, achieving a remarkable accuracy rate of 97.9%.

## 1. Introduction

With the swift progression of new technologies, such as the internet of things (IoT) and artificial intelligence (AI), intelligent manufacturing has emerged as the new norm in the manufacturing sector. Despite this transformation, mechanical machining continues to hold a central role. Research has shown that real-time tool wear identification can enhance machine utilization by 50%, boost productivity by 35% and cut production costs by 30% [1,2,3]. Therefore, the real-time and accurate identification of tool wear state holds paramount significance in enhancing the efficiency of manufacturing processes and the quality of final products.

The identification of tool wear conditions primarily relies on two monitoring approaches: direct and indirect methods. The direct monitoring method utilizes industrial cameras to directly observe the wear state of the tool, ensuring accurate assessments. However, it is susceptible to interference from cutting fluids and chips, and it requires the machine tool to be stopped during measurement, limiting its practical application [4]. In contrast, the indirect monitoring method uses sensor data from the machining process. It establishes a mapping relationship with tool wear. This method provides online monitoring and aligns well with real-time production needs [5]. The indirect monitoring method involves the stages of signal acquisition and preprocessing, feature extraction, feature selection and identification model development.

Direct utilization of the acquired signal data can introduce noise, potentially leading to misinterpretations [6]. Therefore, data preprocessing, especially the employment of denoising techniques, is important to ensure the accurate identification of the tool wear state. After denoising, to avoid directly processing the substantial signal data and to mitigate the risk of overfitting and poor generalization in the identification model, numerous features characterizing tool wear are extracted from the denoised signal [7]. However, not every feature is invariably sensitive to tool wear, necessitating the selection of extracted features.

Recursive feature elimination (RFE) is one of the commonly used feature selection approaches in machine learning. It can select a high-quality feature set and remove redundant and irrelevant features from the dataset [8]. RFE is widely used for machine health diagnosis, prediction, product defect detection and other manufacturing applications [9,10,11]. In the intricate scenarios of tool wear, the strength of RFE lies in its ability to account for interdependencies among features and progressively eliminate the least significant ones. Compared to basic linear correlation methods, RFE is more adept at uncovering complex relationships with the target variable, thereby selecting a more representative feature subset.

Selected signal features can be utilized as inputs, and a tool wear state identification model can be developed. Typical machine-learning models for tool wear identification encompass the artificial neural network (ANN), support vector machine (SVM), hidden Markov model (HMM) and random forest (RF), among others [12]. Cao et al. [13] introduced a tool condition monitoring approach integrated with a field fiber optic temperature measurement device, where spectral features were extracted and input into an ANN for tool state classification. Experimental results showed accuracy consistently above 90% during variable parameters. Basil et al. [14] harnessed sensors to capture vibration data from lathes, employing the random forest algorithm to develop a real-time tool wear classification model, which exhibited notable classification prowess. However, these algorithms are prone to overfitting when processing small sample data. Moreover, since they predominantly rely on the empirical risk minimization principle for optimization, they are susceptible to falling into local optimum solutions, undermining the model’s accuracy and stability.

SVM fundamentally adheres to the structural risk minimization principle, effectively mitigating the risk of overfitting by incorporating regularization terms to control the model’s complexity. Moreover, studies highlight that the non-linear relationship between tool wear and monitoring signals, along with the limited training samples for model development, stand as two significant challenges in tool wear identification [15]. Given SVM’s theoretical foundation in non-linear mapping and its efficacy in small sample machine learning, SVM has found widespread application in the field of tool wear state recognition [16,17,18]. Nonetheless, the efficacy of SVM is significantly influenced by the selection of the penalty factor C and the kernel parameter γ, which directly dictate the model’s classification accuracy and generalization ability. Hence, to fully exploit the potential of SVM grounded in the structural risk minimization principle, it is vital to aptly optimize the penalty factor C and the kernel parameter γ [19].

In recent years, the development of intelligent optimization algorithms has progressed significantly, and researchers have designed algorithms inspired by some natural phenomena, such as gray wolf optimization (GWO) [20], the whale optimization algorithm (WOA) [21], sparrow search algorithm (SSA) [22], northern goshawk optimization (NGO) [23], and so on. These algorithms have been extensively utilized for parameter search optimization in SVM. Especially in the field of tool wear state identification, they play a key role in the training process of identification models. Stalin et al. [24] introduced a tool wear prediction method, leveraging particle swarm optimization (PSO) for SVM tuning, and experimentally demonstrated that PSO can effectively optimize SVM parameters to achieve good prediction accuracy. Ying et al. [25] introduced a broaching tool condition monitoring model optimized with GWO for SVM. The experimental results indicate that, compared to PSO-optimized SVM, the GWO-SVM method demonstrates advantages in terms of classification accuracy and optimization time. Gai et al. [26] constructed a SVM classification model optimized with the WOA, designated for tool wear state identification. The approach’s efficiency and practicality were confirmed through milling wear experimentations. These research works underscore the significant potential of intelligent optimization algorithms in enhancing the performance of tool wear state identification approaches. By leveraging the strengths of these algorithms, researchers can monitor and predict tool wear more accurately and robustly. Although these algorithms show great potential in parameter search optimization, ensuring their convergence accuracy and stability in complex problems remains a challenge.

In 2021, Mohammad et al. [23] introduced the northern goshawk optimization (NGO) as an efficient population intelligence optimization algorithm characterized by fast convergence, robustness and high accuracy of operating results. In recent years, NGO has attracted the attention of many scholars. El-Dabah et al. [27] utilized NGO for identifying the parameters of the photovoltaic module’s triple diode model, and the simulation results showed that NGO accurately extracted the model parameters with superior convergence rate and precision compared to alternative algorithms. Xu et al. [28] developed a northern goshawk optimization–backpropagation artificial neural network (NGO-BP) model for forecasting blood concentration and pharmacokinetic parameters of MET306. The NGO has been successfully utilized in resolving a variety of engineering optimization problems, but how to further improve its convergence accuracy and speed is one of the issues, which this study attempts to address.

Building on the aforementioned research, we use an improved northern goshawk optimization algorithm to optimize the SVM’s penalty factor *C* and kernel parameter *γ* for tool wear state identification. First, the force, vibration and acoustic emission signals are gathered during the milling process. Next, to fully depict the correlation between the signals and tool wear, 245 features from the time, frequency and time–frequency domains are extracted from seven signal channels, forming the initial feature set. Third, to minimize the model’s runtime and data storage requirements while avoiding overfitting, the SVM-RFE model is utilized for feature selection, selecting the optimal feature set most closely related to tool wear. Fourth, the NGO is improved and applied for the first time to the parameter finding of SVM. Ultimately, the optimal feature set is input into the INGO-SVM model for training and prediction, achieving precise tool wear state identification. The feasibility and effectiveness of the proposed approach were validated using the Prognostic and Health Management Society Conference Data Challenge (PHM 2010) real-world dataset [29]. Experimental results show that the method effectively screens features related to tool wear and exhibits strong learning ability to accurately identify tool wear state, achieving an identification accuracy of 97.9% with small sample data. This offers a novel approach for research on tool wear state identification.

The rest of this paper is organized in the following manner. Section 2 offers an in-depth explanation of the proposed method and briefly examines related theories. Section 3 presents the experimental setup relevant to this paper while providing a detailed discussion of the obtained results. Finally, Section 4 serves as the conclusion of this paper.

## 2. Proposed Methodology

### 2.1. Support Vector Machine Recursive Feature Elimination (SVM-RFE)

SVM-RFE is an SVM-based sequential backward selection algorithm utilized for feature selection. The selected features have complementary characteristics, and in each cycle, the features with the lowest scores are removed. However, this does not imply that the top-ranked features alone can achieve the best classification performance for SVM. Multiple features need to be combined to achieve the optimal classification performance, facilitating the fusion of multi-sensor signal features. SVM-RFE involves the following main steps:

Step 1: Determine the kernel function type to be used in the SVM.

Step 2: Train the SVM model using the initial feature set and calculate the importance score Ks for each feature.

The SVM was originally developed for binary classification problems with linearly separable data. Due to the limited scope of the paper, the classification principle of the SVM is not elaborated here. In this paper, the square of the weight vector of the optimal hyperplane of the SVM, i.e., *ω*^2^, is used as the ranking criterion for each feature [30,31]. However, the problem of identifying tool wear state typically involves multiple wear classes, which requires a multi-classification strategy. Therefore, a one-vs-one (OVO) strategy is used, where each category constructs a binary subproblem with all other categories, and if *a* is the number of categories, resulting in A=a(a−1)/2 subproblems. During each SVM-RFE training process, *A* subproblems need to be solved to obtain *A* ranking criterion scores. The *A* ranking criterion scores are then summed to obtain the total score, i.e., $K_{s}=\sum_{i=1}^{A}\omega_{i}^{2}$, which is used as the criterion for feature ranking.

Step 3: Arrange the importance scores of all features in decreasing order and eliminate the feature with the lowest score.

Step 4: Continue iterating Steps 2 and 3 until the remaining features meet the feature reduction criteria.

### 2.2. Northern Goshawk Optimization

#### The Principle of NGO

The hunting strategy of the northern goshawk can be divided into two steps: detecting the prey, and pursuing and evading. The mathematical model formulated by NGO, inspired by these distinct hunting steps, is detailed below:

(1) Prey detection step (exploration step).

In the initial step of the northern goshawk’s hunting process, it randomly chooses the prey and quickly launches an attack. The mathematical representation of the northern goshawk’s behavior in this step is as follows:

(1) $$P_{i}=X_{k},\;i=1,2,\cdots ,N;\;k=1,2,\cdots ,i-1,\cdots ,N,$$

(2) $$x_{i,j}^{new,p1}=\{\begin{array}{c} x_{i,j}+r\left(p_{i,j}-Ix_{i,j}\right),\;F_{P_{i}}<F_{i},\\ x_{i,j}+r\left(x_{i,j}-p_{i,j}\right),\;F_{P_{i}}\geq F_{i}, \end{array}$$

(3) $$X_{i}=\{\begin{array}{cc} X_{i}^{new,p1}, & F_{i}^{new,p1}<F_{i},\\ X_{i}, & F_{i}^{new,p1}\geq F_{i}, \end{array}$$

In this equation, *P_i_* represents the prey’s position selected by the *i*th northern goshawk; $F_{P_{i}}$ represents the objective function value (i.e., the fitness value) of the prey’s location corresponding to the *i*th northern goshawk; *k* is a randomly chosen integer from [1, *N*]; $X_{i}^{new,p1}$ represents the new position of the *i*th northern goshawk; $x_{i,j}^{new,p1}$ represents the new position of the *i*th northern goshawk in the *j*th dimension; $F_{i}^{new,p1}$ represents the fitness value based on the update of the *i*th northern goshawk following this step; *r* is a randomly generated value within [0, 1]; and *I* is a random integer of 1 or 2.

(2) Pursuit and fleeing step (development step).

After being attacked by the northern goshawk, the prey will attempt to flee. During the pursuit, northern goshawks are extremely fast and can catch their prey in various scenarios. Assuming the hunt takes place within a range of attack radius *R*, the mathematical representation of the northern goshawk’s behavior in this step is as follows:

(4) $$x_{i,j}^{new,p2}=x_{i,j}+R\left(2r-1\right)x_{i,j},$$

(5) $$R=0.02\left(1-\frac{t}{T}\right),$$

(6) $$X_{i}=\{\begin{array}{cc} X_{i}^{new,p2}, & F_{i}^{new,p2}<F_{i},\\ X_{i}, & F_{i}^{new,p2}\geq F, \end{array}$$

In this equation, *t* represents the current iteration count, and *T* represents the maximum iteration limit. $X_{i}^{new,p2}$ represents the new position of the *i*th northern goshawk in the second step, while $x_{i,j}^{new,p2}$ represents the new position of the *i*th northern goshawk in the *j*th dimension during the second step, and $F_{i}^{new,p2}$ corresponds to the fitness value based on the update of the *i*th northern goshawk following this step.

### 2.3. Improvement of NGO (INGO)

The NGO has been widely used due to its high convergence accuracy and good robustness. However, it still has certain limitations:

1. During the population initialization step, the NGO employs a method, which generates the initial population randomly. This method results in a high degree of randomness and uneven distribution within the initial population, with individuals exhibiting significant disparities. This can easily lead to a lack of diversity in the population, potentially missing out on potential optimal solutions.
2. In the prey recognition step, the NGO relies heavily on two random numbers, “*r*” and “*I*”, to depict the random behaviors within the population. This excessive randomness might lead to unstable output results, thereby diminishing the quality of solutions.
3. As indicated in Equation (6), the greedy selection mechanism (GSM) governs the population position updates during the pursuit and evasion phases, which easily leads the algorithm into local optima traps.

**Figure 1:**
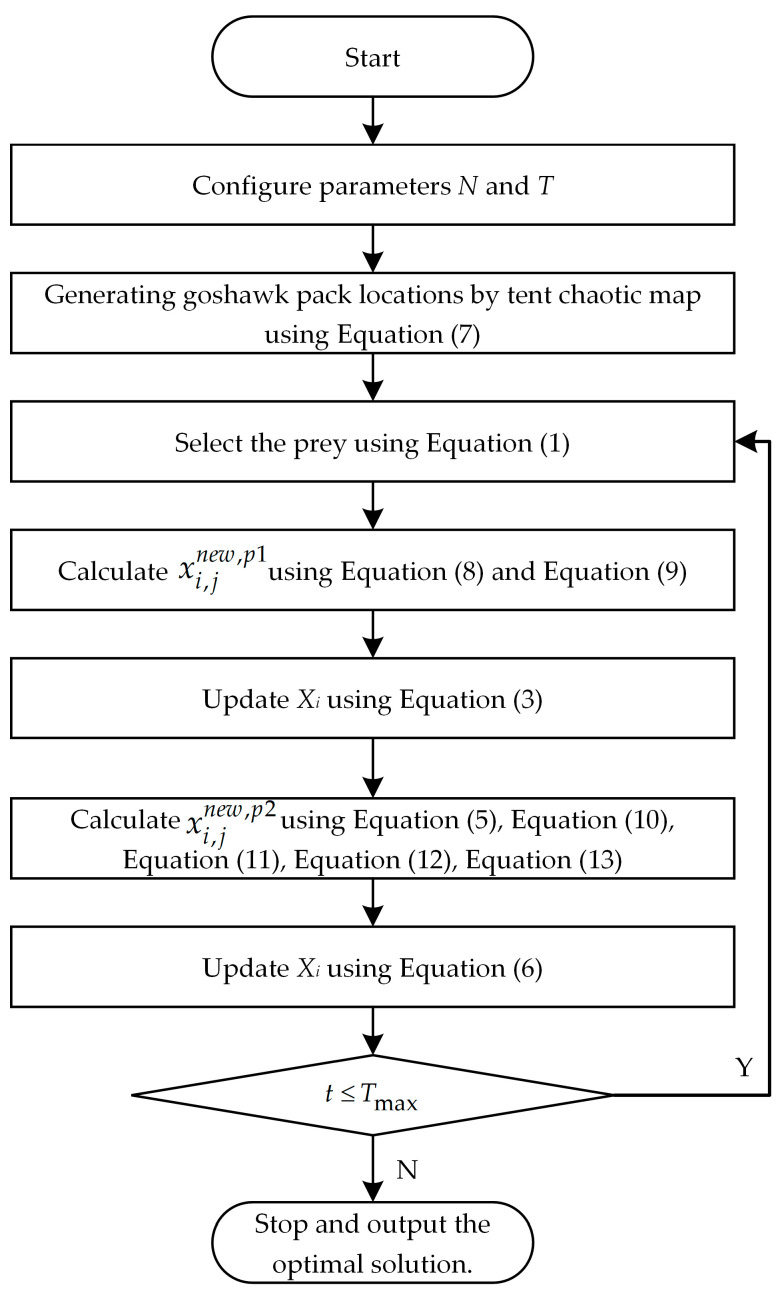
The flowchart of INGO.

Based on the aforementioned analysis, in order to further enhance the optimization capabilities of the NGO, a new method termed INGO has been proposed. Initially, the population is initialized through tent chaos mapping—a process, which not only amplifies the diversity within the population but also facilitates the algorithm in identifying potential optimal solutions from a broader solution space, thereby augmenting its global search capabilities. Subsequently, an adaptive weight factor is introduced during the prey detection step of the NGO to dynamically adjust the search strategy. This adaptive weight factor is capable of automatically modulating the search strategy based on the progression of iterations, consequently reducing the algorithm’s randomness to a certain extent. In the pursuit and fleeing step, we incorporate a Levy flight strategy—a tactic, which renders the algorithm more flexible and diversified during the search process, effectively circumventing premature convergence to local optima. The improved algorithm flowchart is illustrated in Figure 1, and the mathematical principles of the enhanced strategy are as follows:

#### 2.3.1. Tent Chaos Mapping

Chaos mapping is especially adept at initializing populations in optimization algorithms; by substituting random parameters with chaos mapping, the algorithm is capable of generating initial solutions with excellent diversity within the search space [32]. Utilizing the random chaotic sequences generated by tent chaos mapping facilitates the creation of the initial generation of the population. The universal formulation of tent chaos mapping is as follows:

(7) $$x(n+1)=\{\begin{array}{cc} x(n)/\alpha , & x(n)\in [0,\alpha ),\\ (1-x(n))/(1-\alpha )), & x(n)\in [\alpha ,1], \end{array}$$

where α∈[0,1].

#### 2.3.2. Adaptive Weight Factor

During the prey detection step, we introduced a dynamically varying adaptive weight factor, *ω*(*t*), which changes according to the iteration count. In the early stages of iteration, *ω*(*t*) is set to a relatively high value, aiming to amplify the global search capability of the algorithm. As the iteration progresses, *ω*(*t*) will gradually decrease to 0.5, thereby enhancing the algorithm’s local search ability. This strategy assists in maintaining a balance between the global and local search capabilities of the algorithm, ultimately improving the convergence accuracy. The mathematical representation of the adaptive weight factor *ω*(*t*) is as follows:

(8) $$\omega (t)=1-\frac{t}{2\cdot t_{\max}}$$

where *t* is the current iteration count, and *t_max_* is the maximum number of iterations. Consequently, after incorporating the adaptive weight factor, Equation (2) is updated as follows:

(9) $$x_{i,j}^{new,p1}=\{\begin{array}{c} x_{i,j}+\omega (t)\times r\left(p_{i,j}-Ix_{i,j}\right),\;F_{P_{i}}<F_{i}\\ x_{i,j}+\omega (t)\times r\left(x_{i,j}-p_{i,j}\right),\;F_{P_{i}}\geq F_{i} \end{array}$$

#### 2.3.3. Levy Flight Strategy

The Levy flight originates from the integration of Levy’s symmetric stable distribution, serving as a method to generate special random step lengths. Addressing the issue of random searches, many scholars have incorporated this strategy to enhance algorithms, thereby achieving superior optimization results [33,34]. In this paper, the Levy flight strategy is introduced in the second phase of NGO to prevent the population from falling into local optima. The step length of Levy flight follows a heavy-tailed exponential probability distribution (Levy distribution), which adheres to the distribution formula with a step length of *s*:

(10) $$Levy(s)∼u=t^{-1-\beta },\beta \in (0,2]$$

The step equation for the Levy flight process simulation is shown in Equation (11):

(11) $$s=u/|v{|}^{1/\beta }$$

where *β* = 1.5 [35]; *u* and *v* follow a normal distribution with $N\left(0,\delta_{u}^{2}\right)$ and $N\left(0,\delta_{v}^{2}\right)$, respectively. The expressions for $\delta_{u}^{2}$ and $\delta_{v}^{2}$ are as follows:

(12) $$\left\{ \begin{array}{l} \delta_{u}={\left[\frac{\Gamma (1+\beta )\cdot (\sin(\pi \beta /2))}{\Gamma ((1+\beta )/2)\cdot \beta \cdot \left(2^{(\beta -1)/2}\right)}\right]}^{1/\beta }\\ \delta_{v}=1, \end{array}\right.$$

where Γ represents the standard Gamma function integration operation.

**Figure 2:**
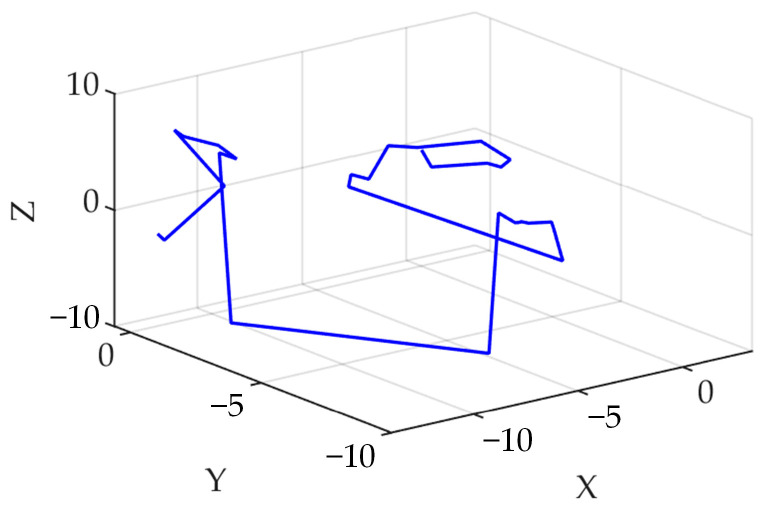
Schematic diagram of the Levy flight in 3D space.

Figure 2 displays a schematic diagram of Levy flight in 3D space, which showcases the random search of the INGO in a 3D space. Equation (4) is transformed by adding the Levy flight strategy:

(13) $$x_{i,j}^{new,p2}=Levy⊗x_{i,j}+R\left(2r-1\right)x_{i,j}$$

where ⊗ is the product of the element.

### 2.4. SVM Parameter Optimization

The INGO algorithm is introduced to search for the penalty factor *C* and kernel function parameter *γ* of the SVM in order to train an optimal identification model. The process of implementing INGO-SVM is outlined below. 

Step 1: Input the training set and test set to establish the fitness function. In this study, the average classification error from five-fold cross-validation serves as the fitness function to evaluate the quality of individual positions, as depicted below:

(14) $$fitness=\frac{1}{K}\sum_{i=1}^{K}\;\left(1-\frac{S{\left(i\right)}^{*}}{S(i)}\times 100\%\right)$$

where *S* is the total number of samples; S* is the number of samples correctly classified by the SVM; and *K* is the K-fold cross-validation, where K=5 in this paper.

Step 2: Initialization of INGO parameters, including the population size *N*, maximum iteration count *T_max_* and the range of optimization for the penalty factor *C* and kernel function parameter *γ*.

Step 3: The initial position of the northern goshawk is initialized using the tent chaos mapping, with individual positions encoded as (C, γ); this ensures a more uniform distribution of the initial population across the parameter range.

Step 4: Conduct iterative optimization following the INGO procedure outlined in Figure 1.

Step 5: Evaluate whether the number of iterations meets the stopping criteria. If not, revert to Step 4. If satisfied, halt the algorithm iteration and output the optimal penalty factor C and kernel function parameter γ, establishing the SVM tool wear state identification model.

**Figure 3:**
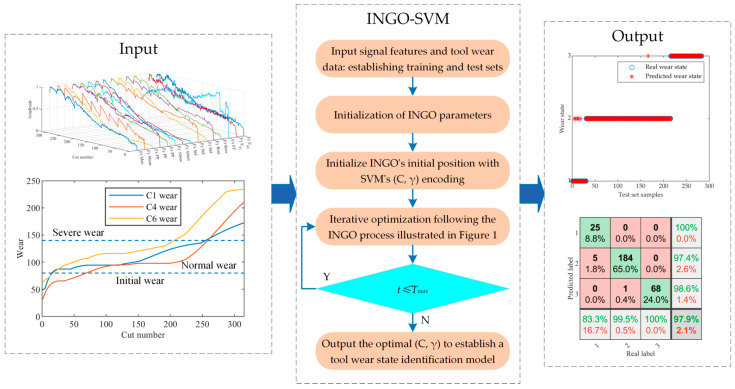
Flowchart of the INGO-SVM tool wear state identification model.

Figure 3 displays the flowchart of the INGO-SVM model.

## 3. Experimental Verification

### 3.1. Performance Testing and Analysis of INGO

#### 3.1.1. Select the Benchmark Test Functions

To validate the optimization performance of INGO, simulation experiments were conducted using eight standard test functions. Functions F1 to F4 are unimodal, assessing the algorithm’s convergence effect, while F5 to F8 are multi-modal, evaluating the algorithm’s local search and search capabilities. Table 1 presents the details of these standard test functions, where n signifies the search dimensionality.

#### 3.1.2. Comparison of INGO with the Other Algorithms

Simulation experiments using standard test functions are performed to confirm INGO’s optimization impact and to compare its performance against NGO and three classic optimization algorithms: PSO, GWO and WOA. For each algorithm, we set the population size to 30 and the maximum number of iterations to 1000, and each standard test function is executed independently 30 times. Table 2 presents the initial parameters for each algorithm.

Simulation experiments were completed using MATLAB R2022b on a computer equipped with an AMD Ryzen 7 5800H CPU, 3.2 GHz base frequency, 32 GB memory and Windows 11 operating system. The mean and standard deviation of the fitness were employed to evaluate the optimization performance. A lower mean fitness value implies increased convergence accuracy, whereas a smaller standard deviation value suggests enhanced algorithm stability. The evaluation formula is expressed as

(15) $$\;Mean=\frac{1}{M}\sum_{i=1}^{M}\;fitness(i)$$

(16) $$Std=\sqrt{\frac{1}{M}\sum_{i=1}^{M}{\left(fitness(i)-Mean\right)}^{2}}$$

where *fitness*(*i*) represents the adaptation value in the *i*th experiment, and *M* is the number of runs for a single experiment.

Table 3 presents the simulation experiment outcomes for each algorithm operating on eight standard test functions, with the bold text denoting the minimum value among all algorithms. The statistical results of INGO for the eight sets of standard test functions outperform significantly those of other comparison algorithms under the same test constraints. For unimodal functions, INGO finds the theoretical optimal values on F1~F3 with a standard deviation of 0. Although the mean value in function F4 is lower, it still outperforms the other algorithms, indicating that INGO has a certain advantage in its ability to seek superiority on unimodal functions. For multi-modal functions, INGO finds the theoretical optimal values on F5 and F7, performing slightly better than GWO, WOA and NGO, while the mean fitness on F8 is slightly lower than that of NGO, ranking second only. In general, INGO exhibits an enhanced capacity to escape local optima on multi-modal functions, and the standard deviation value indicates higher stability for INGO compared to other algorithms. The analysis suggests that the proposed INGO can effectively explore the search space, ensuring robust global and local search capabilities, which significantly improve the algorithm’s convergence accuracy.

In recent years, statistical testing has emerged as a prevalent tool for assessing the performance of computational methods. Particularly in experimental research, they are utilized to observe and compare the performance of different algorithms. Among these, the Wilcoxon signed-rank test has gained favor due to its simplicity in computation and reliability in results [36,37]. To further assess INGO’s performance, a Wilcoxon signed-rank test was conducted on the optimal results of the INGO and four other algorithms over 30 independent runs at a significance level of *p* = 5%, determining whether INGO significantly differed from other intelligent optimization algorithms. The symbols “+”, “=” and “-” represent INGO’s performance as superior, similar or inferior to the comparison algorithms, respectively, while N/A signifies that the algorithms exhibit similar performance and are not comparable. Table 4 demonstrates that in the context of the eight standard test functions considered, INGO exhibits advantageous performance over PSO on all functions, GWO on seven functions, and WOA and NGO on six functions, highlighting its promising capabilities in these instances. Moreover, the majority of *p*-values fall below 5%, signifying that INGO is generally significantly different from the comparison algorithms.

**Figure 4:**
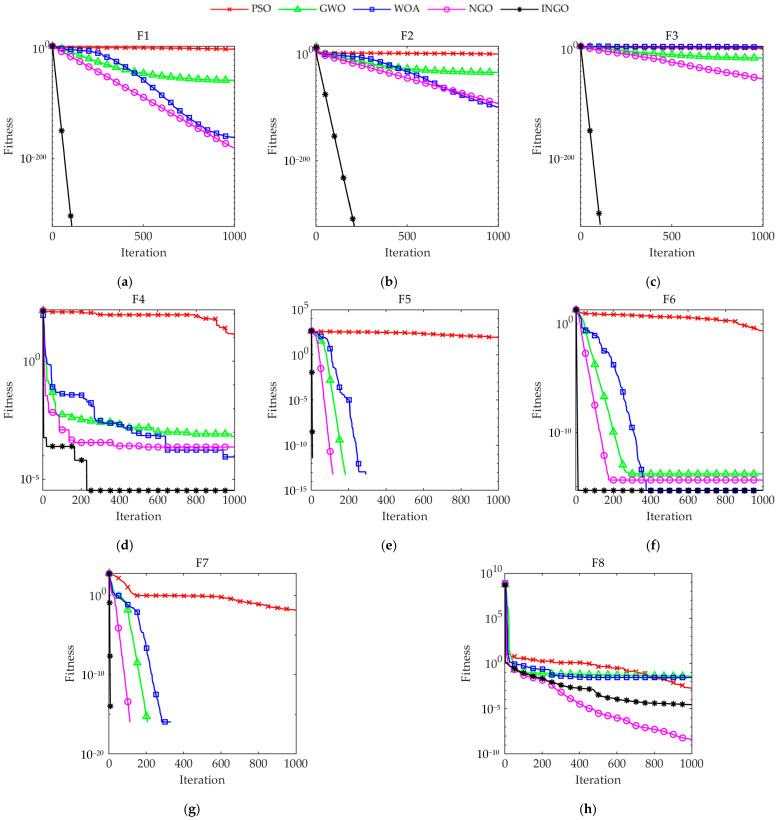
Convergence curves of each algorithm: (**a**) F1; (**b**) F2; (**c**) F3; (**d**) F4; (**e**) F5; (**f**) F6; (**g**) F7; (**h**) F8.

To visually compare the convergence performance of each algorithm, a convergence curve comparison is constructed by choosing the run closest to the average result value, with the horizontal axis representing the iteration count and the vertical axis representing the fitness value. Figure 4 demonstrates that, for various types of test functions, INGO’s convergence speed and accuracy are only slightly lower than NGO’s on F8, while they are significantly superior to other algorithms on the remaining functions. INGO requires the fewest iterations to converge to the same accuracy among the different algorithms. In particular, for F5 and F7, INGO converges to the optimal value in fewer than ten iterations, indicating that the three improvement strategies introduced in this paper effectively enhance the algorithm’s convergence speed and accuracy. However, further evaluation of the performance of INGO in the tool wear state identification problem is still necessary.

### 3.2. Tool Wear Experiment

#### 3.2.1. Description of Experiment

The experiment was designed to validate the proposed method using the real-world PHM 2010 dataset. For the experimental setup, a square stainless steel workpiece with a hardness of HRC52 was subjected to end-milling on a Röders Tech RFM760 computer numerical control (CNC) machine using a ball-ended tungsten carbide cutter. The machining parameters were set as follows: a spindle speed of 10,400 r/min, a feed rate of 1555 mm/min and cutting depths of 0.125 mm radially (Y direction) and 0.2 mm axially (Z direction).

**Figure 5:**
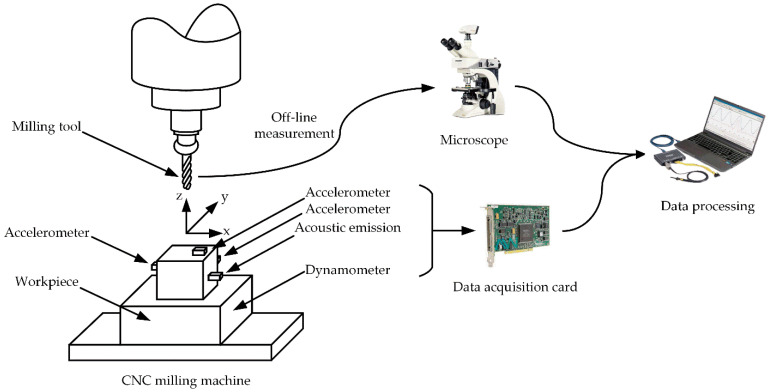
Schematic diagram of the experimental setup.

A three-way force gauge was positioned between the workpiece and the machining table to measure the cutting force. Additionally, vibration signals were captured from three directions using accelerometers, while acoustic waves were recorded by an acoustic emission sensor mounted on the workpiece. Data were collected across seven signal channels at a 50 kHz sampling frequency using the NI PCI 1200 data acquisition card. A detailed schematic representation can be found in Figure 5. More detailed information about the experimental equipment can be found in Table 5.

The experiment involved six milling cutters, labeled C1 through C6. The exact wear values for C2, C3 and C5 were not disclosed; hence, this study primarily focused on data from cutters C1, C4 and C6. Each of these cutters was used 315 times under identical machining conditions. Following each use, the wear values were measured offline on the three flutes of the cutter using a LEICA MZ12 microscope. Specifically, according to ISO 3685:1993 [3], tool wear is determined by measuring the flank wear width (VB) at a depth equal to half of the cutting depth. To best represent authentic machining conditions, the highest wear among the three flutes was considered as the final metric for each cutter. For detailed analysis, 100,000 sample points from the center of each milling operation were selected, excluding anomalies during the cutter’s entry and exit. The experimental equipment and machining parameters are shown in Table 5.

#### 3.2.2. Performance of the Proposed Methodology

During the milling process, the collected vibration signal contains a significant quantity of noise. In order to reliably and effectively assess the identification tool wear state, it is necessary to perform denoising on the signal. Wavelet packet threshold denoising (WPTD) has demonstrated great advantages in denoising non-stationary signal and is widely used for this purpose [38]. WPTD involves three parts: decomposition, threshold denoising and reconstruction, which essentially filters the signal. In this paper, the “db3” wavelet basis function is selected to perform four-level wavelet packet decomposition and denoise the vibration signal, decomposing the signal into 16 frequency intervals of different frequency bands. The unbiased risk estimation threshold (rigrsure) rule is used to select the threshold value to distinguish between noise and noiseless signal. The noise is then filtered out by the soft threshold function, and the noiseless signal is reconstructed to achieve denoising [39,40].

After denoising, the wavelet packet energy was extracted across the entire frequency band (1~25 kHz) from all seven signal channels. For each channel, 16 time–frequency domain features were obtained through a four-level wavelet packet decomposition. Additionally, 14 time–domain features and 5 frequency domain features were extracted from each channel. Thus, per channel, we had 16 + 14 + 5 = 35 features. With data from seven channels, the total number of extracted features was 35 × 7 = 245 features. The types of extracted features are presented in Table 6.

To minimize the model’s running time and required data storage space, as well as to prevent overfitting, irrelevant or redundant features were eliminated using SVM-RFE. To normalize the extracted features, we utilized the max–min normalization method. Assuming $\bar{x}$ denotes the normalized data and *x_i_* denotes the original data, the equation is as follows:

(17) $$\bar{x}=\frac{x_{i}-\min\left\{x_{i}\right\}}{\max\left\{x_{i}\right\}-\min\left\{x_{i}\right\}}$$

The LIBSVM toolbox is employed to construct the SVM base model [41]. Given that the dataset we utilize exhibits characteristics such as small sample size, high dimensionality and non-linearity, the kernel function selected for this paper is the radial basis function (RBF), represented by the equation below:

(18) $$\left\{ \begin{array}{l} K\left(x,x_{i}\right)=\exp\left\{-\frac{{\left|x-x_{i}\right|}^{2}}{\gamma }\right\},\\ \gamma =\frac{1}{2\sigma^{2}}, \end{array}\right.$$

**Figure 6:**
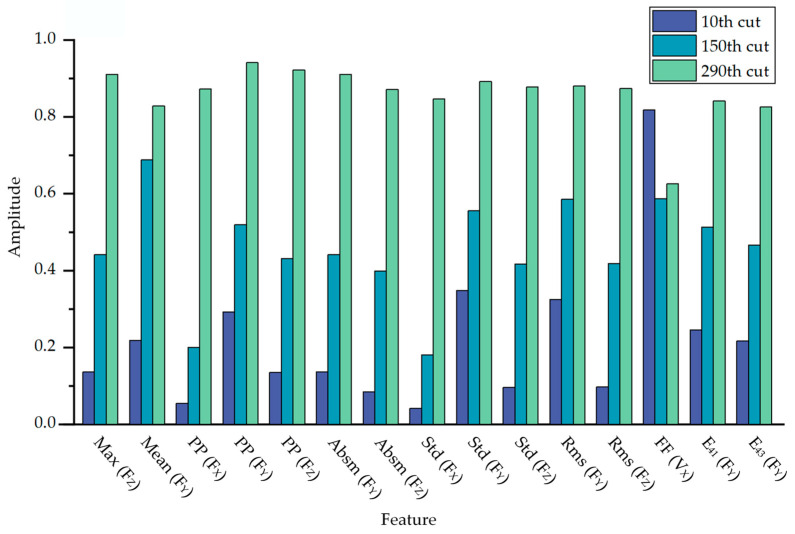
Comparison of feature amplitudes at different wear states in C1.

**Figure 7:**
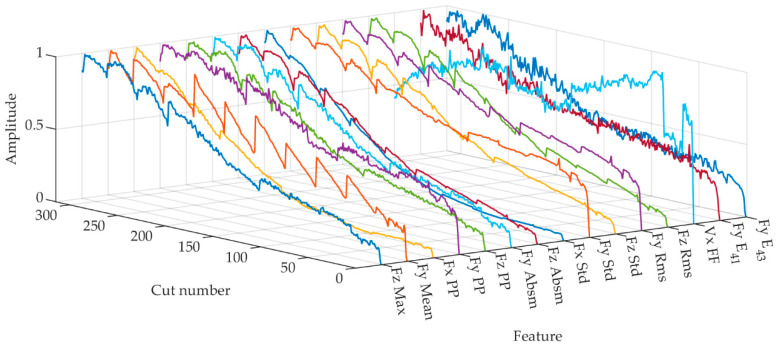
Normalized features in C1.

Taking into account the real-time monitoring requirements, we retained 15 features to form the optimal feature set. Table 7 presents the final set of optimal features, where E_41_ and E_43_ represent the WPE of the second and fourth frequency bands. Exemplified using milling cutter C1, we selected the amplitudes of these features during the 10th (initial wear), 150th (normal wear) and 290th (severe wear) milling pass for comparison, as depicted in Figure 6. Concurrently, Figure 7 displays the variation trends of these 15 features after normalization.

According to Figure 6 and Figure 7, we observed that in all three cutting force directions, there exist optimal features represented by both PP and Std. These parameters delineate the difference between signal peaks and troughs, and the dispersion or fluctuation of the signal, respectively. PP potentially characterizes the maximum cutting force exerted on the tool during the machining process. As the wear progresses, the cutting edge of the tool becomes less sharp, enlarging the cutting area and necessitating greater force for material removal, leading to an augmentation in PP. On the other hand, a rising Std suggests irregular fluctuations in the cutting force. Tool wear intensifies the instability in the cutting process. For instance, due to wear at the tool tip, there might be heightened vibrations during machining, amplifying force oscillations. 

**Figure 8:**
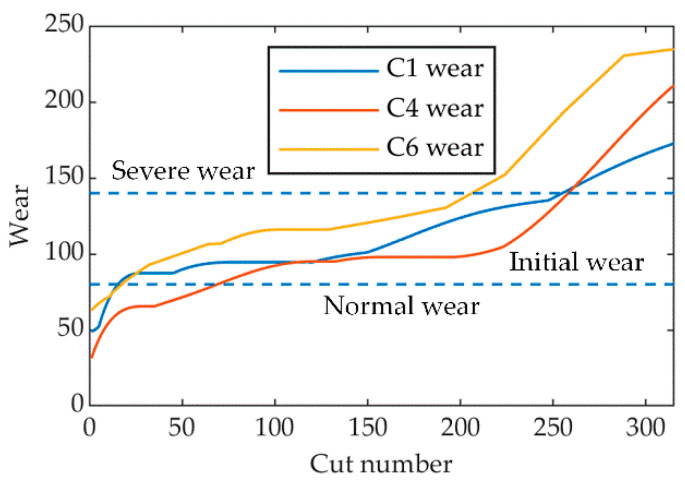
Tool wear curves of C1, C4 and C6.

According to Figure 6, a notable observation was the pronounced increase in both PP and Std between the 150th and 290th cycle, surpassing the growth observed from the 10th to the 150th cycle. This shift is attributed to the tool’s evident morphological changes during its intense wear phase, causing notable increases in force and process irregularities. We also noted some vibration signal features correlating with tool wear. However, when employing SVM-RFE for optimal feature selection, we chose the top 15 features most sensitive to tool wear, aiming for a model, which is both efficient and concise. The features selected predominantly originate from cutting force signals, for, under the prescribed experimental setup, they present a more direct and sensitive metric for tool wear than vibration signals. Simultaneously, with an increase in the number of milling cycles, the optimal features generally exhibit an upward trend, which is largely consistent with the wear trend of milling tools shown in Figure 8. This also suggests that SVM-RFE can effectively select the features most sensitive to tool wear.

Based on the existing literature, tool wear can be categorized into three states: initial wear state (0–80 μm), normal wear state (80 μm–140 μm) and severe wear state (140 μm–∞) [3,42,43]. Figure 8 displays the wear curves of the three milling tools, leading to the sample size distribution for each category, as depicted in Table 8. An optimal set of features of the three milling cutters and their corresponding label categories were formed into a dataset. Samples from each category were divided into training and testing sets at a ratio of 0.7:0.3. The dataset was then input into the INGO-SVM identification model to evaluate the identification performance of our proposed approach.

**Figure 9:**
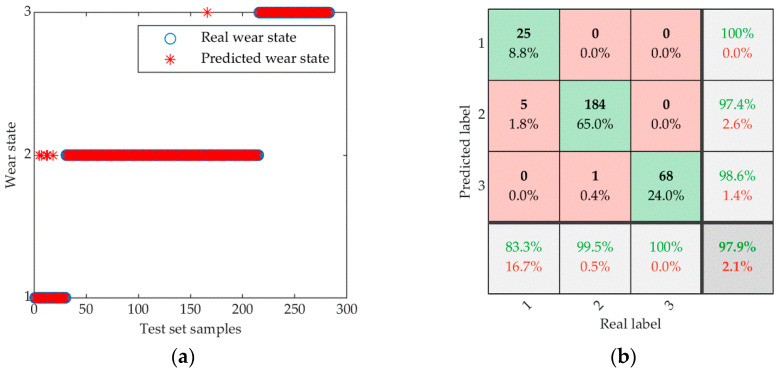
Identification results of INGO-SVM: (**a**) Identification results of wear state; (**b**) Confusion matrix.

Relying solely on a single accuracy rate to evaluate the identification outcomes may be limited, given the significant disparity in sample sizes among the three categories in the test set. Thus, to appraise the INGO-SVM’s performance, we employ four evaluation metrics: accuracy, precision, recall and macro mean (F1-score). Table 9 presents the approach’s evaluation results on the test set, while Figure 9 displays the identification outcomes and confusion matrix of INGO-SVM.

As shown in Figure 9, our proposed approach achieves an overall identification accuracy of 97.9%, with identification accuracies of approximately 83.3%, 99.5% and 100% for the three wear states, respectively. It is worth noting that tool failure usually occurs in the severe wear state, and INGO-SVM performs better in this state. Hence, the tool wear identification approach presented in this study exhibits exceptional accuracy and dependability.

#### 3.2.3. Comparison and Discussion

**Figure 10:**
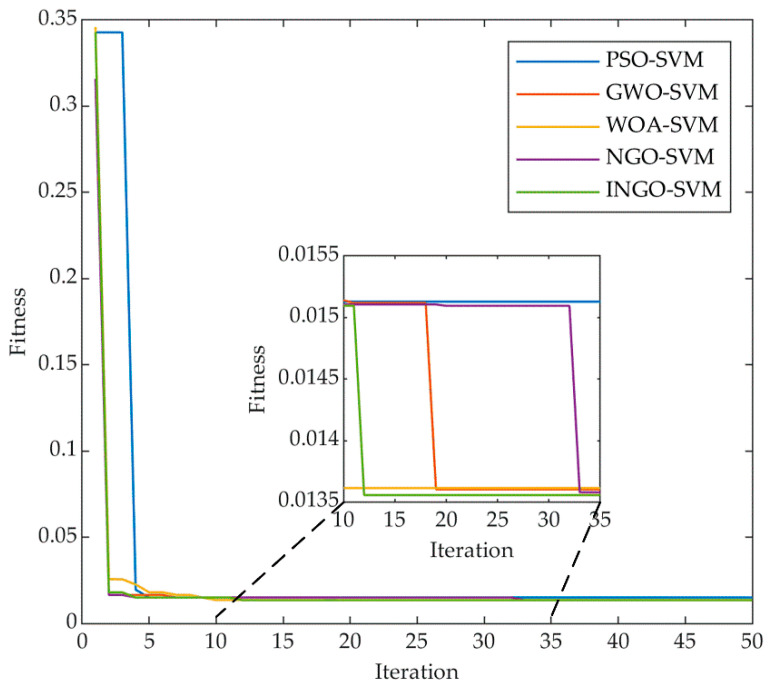
Convergence curves of five identification approaches in the training set.

In this paper, we compare INGO-SVM with PSO-SVM [24], GWO-SVM [25], WOA-SVM [26], NGO-SVM and unoptimized SVM using the same data. For each algorithm, the population size was established at 10, and the maximum iteration count was designated as 50. The initial parameter settings for each algorithm are as shown in Table 2. Figure 10 displays the fitness variation curves of the five identification approaches on the training set, while Table 10 presents the wear state identification results on the test set. As shown in Figure 10, all five algorithms converge to a stable state as the number of iterations increases. PSO converges quickly and requires fewer iterations to reach the optimal value, while GWO eventually converges to a better accuracy than PSO but requires more iterations. WOA exhibits similar convergence characteristics to PSO but ultimately converges with the same accuracy as GWO. During the early iterations, the fitness value of NGO changes relatively little and only converges to the same value as GWO at the 33rd iteration. All four algorithms except INGO fall into local optimality, leading to poorer identification results in the test set. In contrast, INGO converges by the 12th iteration and achieves the optimal value among all algorithms, with the optimal value for *C* being 45.981 and the optimal value for *γ* being 0.3861, indicating that INGO possesses faster convergence and higher accuracy in this particular scenario.

As shown in Table 10, all approaches using parametric optimization outperformed the approach without parametric optimization in all four evaluation criteria. Among them, INGO-SVM achieved the highest accuracy (97.9%), precision (98.6%) and F1-score (96.2%) on the test set. The F1-score is more suitable for evaluating industrial datasets with unbalanced samples, such as tool wear, as it combines the two metrics of precision and recall. Furthermore, INGO-SVM attained an average classification accuracy of 98.6% on the training set, which validates the model’s generalization performance. As a result, INGO proves to be a more fitting choice for parameter optimization of the SVM applied to tool wear.

## 4. Conclusions and Discussion

In this paper, we proposed a tool wear state identification model utilizing an improved northern goshawk optimization algorithm to optimize the support vector machine, and we verified its feasibility through the PHM 2010 real-world dataset. The primary research findings are as follows:

(1) The NGO was theoretically enhanced through three key modifications to elevate its solution accuracy and convergence speed. Firstly, the integration of tent chaos mapping in the NGO improved the quality of the initial population. Secondly, the introduction of an adaptive weight factor harmoniously balanced the global and local search capabilities, thereby reducing the randomness inherent in the algorithm. Lastly, the implementation of the Levy flight strategy effectively prevented the algorithm from becoming trapped in local optimum solutions, fostering a more robust optimization process.
(2) Eight benchmark test functions were selected to compare the INGO with PSO, GWO, WOA and NGO algorithms. After conducting 30 simulation experiments, INGO demonstrated superiority in terms of the mean and standard deviation on seven of the functions, showcasing its enhanced optimization performance and stability.
(3) A sophisticated data processing approach was employed, where signals from seven channels underwent a four-layer wavelet packet threshold denoising process. This was followed by the extraction of wavelet packet energy across the entire frequency band, coupled with several statistical features. This meticulous process, facilitated by the SVM-RFE method, enabled the selection of an optimal feature set, setting a solid foundation for the development of a more accurate and reliable tool wear state identification model.
(4) Utilizing INGO to optimize the parameters of SVM, and adopting the average classification error from five-fold cross-validation as the fitness function, the INGO-SVM showcases higher convergence precision and classification accuracy compared to existing identification methods. This approach achieves a wear state identification accuracy rate of up to 97.9%, representing an approximate improvement of 15.6% and 3.2% over SVM and NGO-SVM, respectively, thus demonstrating a notable advantage in the experiments conducted. These results indicate that the INGO-SVM model possesses superior classification accuracy, making it viable for real-time monitoring of tool wear conditions.

The proposed method for tool wear state identification provides an effective approach for real-time tool condition monitoring and early warning in practical machining, showcasing potential application value. However, another crucial aspect of tool state monitoring concerns the prediction of the tool’s remaining useful life. In subsequent research, we anticipate that a more comprehensive solution for tool management will be provided by integrating the INGO algorithm with other advanced predictive technologies.

## Figures and Tables

**Table 1:** Benchmark test functions.

Name	Expression	Dimension	Search Space	Optimal Value
Sphere	$F_{1}(x)=\sum_{i=1}^{n}x_{i}^{2}$	30	[−100,100]	0
Schwefel 2.22	$F_{2}(x)=\sum_{i=1}^{n}\left|x_{i}\right|+\prod_{i=1}^{n}\left|x_{i}\right|$	30	[−10,10]	0
Schwefel 1.2	$F_{3}(x)=\sum_{i=1}^{n}{\left(\sum_{j=1}^{i}x_{j}\right)}^{2}$	30	[−100,100]	0
Quartic	$F_{4}(x)=\sum_{i=1}^{n}ix_{i}^{4}+random[0,1)$	30	[−1.28,1.28]	0
Rastrigin	$F_{5}(x)=\sum_{i=1}^{n}\left[x_{i}^{2}-10\cos\left(2\pi x_{i}\right)+10\right]$	30	[−5.12,5.12]	0
Ackley	$\begin{array}{c} F_{6}(x)=-20\exp\left(-0.2\sqrt{\frac{1}{n}\sum_{i=1}^{n}x_{i}^{2}}\right)-\exp\left(\frac{1}{n}\sum_{i=1}^{n}\cos\left(2\pi_{x_{i}}\right)\right)\\ +20+e \end{array}$	30	[−32,32]	0
Griewank	$F_{7}(x)=\frac{1}{4000}\sum_{i=1}^{n}x_{i}^{2}-\prod_{i=1}^{n}\cos\left(\frac{x_{i}}{\sqrt{i}}\right)+1$	30	[−600,600]	0
Penalized 1	$\begin{array}{c} F_{8}(x)=\frac{\pi }{n}\{10\sin\left(\pi y_{1}\right)+\sum_{i=1}^{n-1}{\left(y_{i}-1\right)}^{2}\\ \left[1+10\sin^{2}\left(\pi y_{i+1}\right)\right]+\sum_{i=1}^{n}u\left(x_{i},10,100,4\right)\}\\ y_{i}=1+\frac{x_{i}}{4}u\left(x_{i},a,k,m\right)=\{\begin{array}{c} k{\left(x_{i}-a\right)}^{m},x_{i}>a\\ 0,-a<x_{i}<a\\ k{\left(-x_{i}-a\right)}^{m},x_{i}<-a \end{array} \end{array}$	30	[−50,50]	0

**Table 2:** Initialization parameters of all algorithms.

Algorithm	Values of the Parameters
PSO	$c_{1}=c_{2}=2$
GWO	*a*:Linear reduction from 2 to 0
WOA	*a*:Linear reduction from 2 to 0
NGO	*r* = [0, 1], *I* = {1, 2}
INGO	*r* = [0, 1], *I* = {1, 2}, α=0.7,β=1.5

**Table 3:** Comparison of the experimental results of each algorithm.

Algorithm	Statistics	Function							
		F1	F2	F3	F4	F5	F6	F7	F8
PSO	Mean	0.2880	0.9667	78.9099	2.8202	106	1.1147	0.0216	0.0059
	Std	0.1395	0.3465	25.4474	1.9699	28.4601	0.5690	0.0105	0.0190
GWO	Mean	2.83 × 10^−58^	1.41 × 10^−34^	2.84 × 10^−14^	8.69 × 10^−4^	0.5376	1.51 × 10^−14^	0.0021	0.0473
	Std	9.54 × 10^−58^	1.55 × 10^−34^	9.56 × 10^−14^	7.80 × 10^−4^	2.1970	2.86 × 10^−15^	0.0050	0.0240
WOA	Mean	1.35 × 10^−80^	6.71 × 10^−39^	3.82 × 10^−31^	0.0011	**0**	3.52 × 10^−15^	0.0023	0.0070
	Std	7.25 × 10^−80^	3.39 × 10^−38^	2.02 × 10^−30^	8.51 × 10^−4^	**0**	2.20 × 10^−15^	0.0123	0.0068
NGO	Mean	1.21 × 10^−179^	1.58 × 10^−92^	2.95 × 10^−48^	2.81 × 10^−4^	**0**	5.54 × 10^−15^	**0**	**3.75 × 10^−9^**
	Std	**0**	3.25 × 10^−92^	1.59 × 10^−47^	1.22 × 10^−4^	**0**	1.76 × 10^−15^	**0**	**2.73 × 10^−9^**
INGO	Mean	**0**	**0**	**0**	**2.11 × 10^−5^**	**0**	**4.44 × 10^−16^**	**0**	2.03 × 10^−5^
	Std	**0**	**0**	**0**	**2.20 × 10^−5^**	**0**	**0**	**0**	2.85 × 10^−5^

**Table 4:** *p*-value of Wilcoxon signed-rank test.

	F1	F2	F3	F4	F5	F6	F7	F8	+/=/−
INGO-PSO	1.73 × 10^−6^	1.73 × 10^−6^	1.73 × 10^−6^	1.73 × 10^−6^	1.73 × 10^−6^	1.73 × 10^−6^	1.73 × 10^−6^	6.34 × 10^−6^	8/0/0
INGO-GWO	1.73 × 10^−6^	1.73 × 10^−6^	1.73 × 10^−6^	1.73 × 10^−6^	4.88 × 10^−4^	5.96 × 10^−7^	0.0300	0.3820	7/0/1
INGO-WOA	1.73 × 10^−6^	1.73 × 10^−6^	1.73 × 10^−6^	1.92 × 10^−6^	N/A	5.79 × 10^−5^	0.2500	0.0039	6/1/1
INGO-NGO	1.73 × 10^−6^	1.73 × 10^−6^	1.73 × 10^−6^	1.73 × 10^−6^	N/A	6.91 × 10^−7^	N/A	6.34 × 10^−6^	6/2/0

**Table 5:** Experimental equipment and machining parameters.

Category	Parameter	Value
Experimental equipment	CNC machine	Röders Tech RFM760
	Three-way force gauge	Kistler 9265B
	Accelerometer	Kistler 8636C
	Acoustic emission sensor	Kistler 8152
	Data acquisition card	NI PCI 1200
	Microscope	LEICA MZ12
Machining parameters	Spindle speed	10,400 r/min
	Feed rate	1555 mm/min
	Cutting depth (Radial, Y direction)	0.125 mm
	Cutting depth (Axial, Z direction)	0.2 mm
Workpiece and Cutter material	Workpiece material	HRC 52
	Cutter material	Ball-ended tungsten carbide cutter

**Table 6:** Extracted features of signal.

Domain	Features
Time domain	Maximum value (Max) Minimum value (Min)
	Mean value (Mean) Peak-to-peak value (PP)
	Absolute mean (Absm) Variance (Var)
	Standard deviation (Std) Kurtosis (Kur)
	Skewness (Ske) Root mean square (Rms)
	Form factor (FF) Crest factor (CF)
	Impulse factor (IF) Margin factor (MF)
Frequency domain	Frequency centroid (FC) Mean square frequency (MSF)
	Root mean square frequency (RMSF) Frequency variance (FV)
	Frequency standard deviation (FSD)
Time–frequency domain	Wavelet packet energy after four-level decomposition (WPE)

**Table 7:** Best features combination.

Signal Channel	Features
$F_{x}$ (Force signal in the X direction)	PP Std
$F_{y}$ (Force signal in the Y direction)	Mean PP Absm Std Rms $E_{41}$ $E_{43}$
$F_{z}$ (Force signal in the Z direction)	Max PP Absm Std Rms
$V_{x}$ (Vibration signal in the X direction)	FF
$V_{y}$ (Vibration signal in the Y direction)	/
$V_{z}$ (Vibration signal in the Z direction)	/
AE (Acoustic emission signal)	/

**Table 8:** Number of samples in each category.

Wear State	Training Set	Test Set	Label
Initial wear	70	30	1
Normal wear	433	185	2
Severe wear	159	68	3

**Table 9:** Identification accuracy of the proposed methodology.

Accuracy	Precision	Recall	F1-Score
97.9	98.6	94.3	96.2

**Table 10:** Results of the six identification approaches in the test set.

Method	Accuracy	Precision	Recall	F1-Score
PSO-SVM	91.2	85.7	93.6	88.4
GWO-SVM	93.3	91.1	88.2	89.2
WOA-SVM	94.0	90.1	89.8	90.0
NGO-SVM	94.7	90.3	96.1	92.4
INGO-SVM	97.9	98.6	94.3	96.2
SVM	82.3	78.8	87.9	79.1

## Data Availability

Experimental data were obtained from the Prognostics and Health Management Society 2010 PHM Society Conference Data Challenge. The resource can be found in the corresponding reference.
